# Extending the Window: A Systematic Review of Pharmacological Adjuncts for Single-Shot Adductor Canal Blocks in Total Knee Arthroplasty

**DOI:** 10.3390/jcm15135005

**Published:** 2026-06-26

**Authors:** Genevieve Crotty, André van Zundert

**Affiliations:** 1 Prince of Wales Hospital, Randwick, Sydney, NSW 2031, Australia; 2 Department of Anaesthesia and Perioperative Medicine, Royal Brisbane and Women’s Hospital, Brisbane, QLD 4029, Australia; 3 Faculty of Medicine, The University of Queensland, Brisbane, QLD 4029, Australia

**Keywords:** adductor canal block, total knee arthroplasty, postoperative analgesia, pharmacological adjuncts, dexmedetomidine, dexamethasone

## Abstract

**Background/Objectives**: Adductor canal blocks (ACBs) are widely used for postoperative analgesia following total knee arthroplasty (TKA). However, the duration of analgesia with a single-shot ACB is limited. Pharmacological adjuncts may enhance and prolong the duration of single-injection blocks, but their efficacy in this setting remains unclear. The aim of this study was to assess the analgesic effectiveness of adjuncts added to local anaesthetic for single-shot ACBs following TKA. **Methods**: An extensive systematic literature review was performed on Medline, Embase, CINAHL, Cochrane CENTRAL, and Web of Science. Adult patients undergoing primary TKA who received a single-shot ACB with an adjunct added to LA were eligible, with a single-shot ACB with LA alone as the comparator. The primary outcome was postoperative analgesic efficacy, assessed by pain scores (VAS/NRS), time to first rescue analgesia, total postoperative opioid consumption, or sensory block duration. Secondary outcomes included functional recovery measures and adverse events. Risk of bias was evaluated using the Cochrane RoB 2 tool, and the certainty of evidence for each adjunct–outcome combination was assessed using GRADE. Due to clinical heterogeneity, a meta-analysis was not feasible and findings were synthesised narratively. **Results**: Nine randomised controlled trials (RCTs) assessing the analgesic efficacy of adjuncts added to local anaesthetic in ACB following TKA were included in this review. Adjuncts included dexmedetomidine, dexamethasone, butorphanol, buprenorphine, and magnesium sulphate. Three RCTs demonstrated improvements in early postoperative analgesia with dexmedetomidine at doses of 0.5 µg/kg, while lower doses (0.25 µg/kg) did not. Dexamethasone also decreased early postoperative pain across two RCTs and showed the most evidence for significant prolongation in sensory blockade, with a dose of 4 mg needed to produce significant effects. Butorphanol and buprenorphine demonstrated a significant reduction in postoperative opioid consumption and improved pain, but evidence was limited to single trials. Findings for magnesium were inconsistent. No adjunct was associated with any serious side effect or adverse event. **Conclusions**: Pharmacological adjuncts added to single-shot ACBs following TKA generally improved early postoperative pain and reduced opioid consumption compared with LA alone, with the most consistent benefits observed for dexmedetomidine (0.5 µg/kg) and dexamethasone (≥4 mg). However, these effects appeared dose-dependent, were largely confined to the first 24 h after surgery, and were supported by moderate-to-low certainty evidence with limited functional outcome data. Further high-quality, adequately powered RCTs with standardised functional endpoints and longer follow-up are required to define optimal dosing, clarify safety, and determine whether improved analgesia translates into meaningful gains in rehabilitation and recovery.

## 1. Introduction

Total knee arthroplasty (TKA) is a common orthopaedic procedure to manage severe knee pain, generally caused by osteoarthritis. This major surgical procedure is often accompanied by significant postoperative pain [1]. Inadequate pain control may delay early mobilisation and rehabilitation, which can increase the risk of postoperative complications and negatively impact recovery. Therefore, an effective analgesic strategy needs to be implemented to optimise patient outcomes.

Regional anaesthesia is an integral component of pain management after TKA, as it provides effective pain relief while minimizing the use of opioids [2]. Historically, the femoral nerve block was the preferred regional anaesthetic technique for TKA. However, adductor canal blocks (ACBs) have gained prominence as an alternative method due to their preservation of quadriceps motor function. The adductor canal contains primarily sensory nerves, including the saphenous nerve, while largely sparing the motor branches of the femoral nerve that innervate the quadriceps muscle [3]. As a result, ACBs preferentially produce sensory blockade with minimal motor impairment [3]. This distinction allows for preservation of quadriceps strength, facilitating earlier mobilisation and better short-term functional outcomes, as consistently reported in the literature [4,5].

ACBs can be performed as a single-injection or a continuous catheter technique. Continuous catheters have previously been the preferred technique to extend analgesia, given their ability to deliver ongoing local anaesthetic as an infusion, providing analgesia beyond the typical duration of a single-shot block. However, recent research suggests no analgesic benefit over single-shot ACBs, while introducing catheter-related burdens [4,5]. Continuous catheters require additional equipment, skilled nursing staff, and dedicated follow-up. They also introduce risks associated with catheter placement, such as infection and dislodgement [6]. For these reasons, attention has shifted toward pharmacological adjuncts to enhance and prolong the duration of single-injection blocks as a simpler alternative.

Numerous studies have shown that adjuncts like dexmedetomidine, dexamethasone, buprenorphine, butorphanol and magnesium sulphate can extend analgesia when added to local anaesthetic (LA) in peripheral nerve blocks [7,8,9,10,11,12,13]. Despite a growing body of evidence supporting adjuncts in peripheral nerve blockade, their analgesic efficacy when added to LA in single-shot ACBs has not been confirmed. This review aimed to assess the analgesic effectiveness of pharmacological adjuncts added to local anaesthetic for single-shot ACBs following TKA.

## 2. Methods and Materials

**Figure 1:**
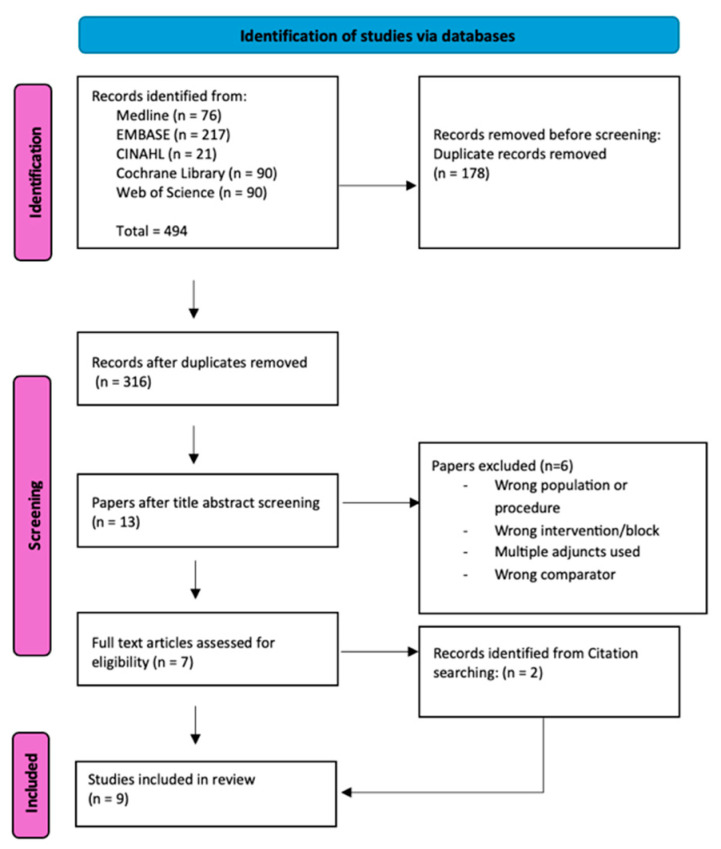
PRISMA identification, screening and selection of articles.

We conducted this systematic review in accordance with the Preferred Reporting Items for Systematic Reviews and Meta-Analyses (PRISMA) guidelines and prospectively registered the study in PROSPERO (Registration Number: CRD420261384962). The study selection process is summarised in the PRISMA flow diagram (Figure 1). The objective was to assess the analgesic efficacy of adjuvants added to local anaesthetic in adductor canal block (ACB) for postoperative analgesia following total knee arthroplasty (TKA). Generative AI (Perplexity, powered by GPT-5.1, Perplexity AI) was used to assist with identifying supplementary literature and for language editing of draft sections. All content was checked and verified by the authors, who take full responsibility for the final manuscript.

### 2.1. Search Strategy

A comprehensive literature search was conducted in the following databases: Medline (Ovid), Embase (Elsevier), CINAHL (EBSCO), Cochrane CENTRAL, and Web of Science (Clarivate). No restrictions were applied with respect to publication date, language, or study type. Conference abstracts were removed from the Embase search results, and records originating from clinical trial registries were excluded from CENTRAL. Search results were exported to EndNote 2025, where duplicate and non-English records were identified and removed.

### 2.2. Eligibility Criteria

Eligibility criteria were defined using the Population, Intervention, Comparison, Outcomes, and Study design (PICOS) framework. The population included adult patients (≥18 years) undergoing primary total knee arthroplasty (TKA). The intervention was a single-shot ACB with a pharmacological adjunct added to the local anaesthetic (LA). The comparator was a single-shot ACB with LA alone. The outcome measured was postoperative analgesia following TKA. Studies were eligible for inclusion if they directly compared single-shot ACB with adjuncts versus single-shot ACB without adjuncts. Exclusion criteria included non-English studies, paediatric populations, use of catheters, or patients undergoing TKA with peripheral regional anaesthesia that did not include ACB. Randomised controlled trials, prospective cohort studies, and relevant observational studies were considered. Review articles, scoping reviews, protocols, editorials, letters, and case reports were excluded.

### 2.3. Study Selection

The remaining records were screened in two stages: initial title and abstract screening, followed by full-text review to determine eligibility. In addition, the reference lists of all included studies were manually screened to identify any further eligible articles not captured in the initial database search. Studies meeting the predefined inclusion criteria were included in the final synthesis. The study selection process is summarised in the PRISMA flow diagram (Figure 1).

### 2.4. Primary and Secondary Outcomes

The primary outcome was postoperative analgesia, measured using patient-reported pain scores (VAS or NRS), time to first rescue analgesia, total postoperative opioid consumption at predefined times, or objective assessment of sensory block duration. Secondary outcomes included functional recovery measures and adverse events.

### 2.5. Risk of Bias Assessment

The methodological quality of included studies was assessed using the Cochrane Risk of Bias 2 (RoB 2) tool, shown in Table 1. Two studies were judged to be at high overall risk of bias due to the absence of prospective trial registration (Domain 5). The remaining seven trials raised some concerns related to ambiguity around allocation concealment and the potential for selective outcome reporting. Overall, these findings suggest a moderate risk of bias, and findings from included trials should be interpreted with caution.

### 2.6. Data Synthesis and GRADE

Due to clinical and methodological variability in outcome measurement across the studies, a meta-analysis was not feasible. The primary sources of heterogeneity were local anaesthetic variability (varying by agents and volumes), ACB timing (pre-operative vs. post-operative), background anaesthetic techniques and multimodal analgesic protocols, and non-uniform outcome definitions and timepoints for pain scores, opioid consumption, and time to first rescue. Additionally, opioid outcomes were measured using varying drugs and units (e.g., morphine equivalents, hydrocodone equivalents, oxycodone equivalents, PCIA attempts) and reported within different timeframes and under different conditions (rest vs. movement). Because the studies included used different drugs, doses, time periods, analgesic techniques and outcome measures, combining their results would have required many assumptions and the pooled results would not reflect a clearly defined clinical setting. Instead, a narrative summary of the results was performed, where the findings were categorised based on adjunct type and outcomes. The certainty of evidence for each adjunct-outcome combination was therefore assessed using the GRADE (Grading of Recommendations, Assessment, Development and Evaluation) framework. This framework evaluates randomised controlled trial evidence across five domains: risk of bias, inconsistency, indirectness, imprecision, and publication bias. Each comparison begins at HIGH certainty (as evidence derives from RCTs) and may be downgraded one or more levels to a final rating of high, moderate, low or very low. High certainty in evidence means that the investigators are very confident that the effect they found across studies is close to the true effect, and very low means that thedy have very little confidence in the effect [23]. Because no meta-analysis was performed and each comparison is supported by a small, heterogeneous evidence base, these GRADE ratings should be interpreted as preliminary and used as supportive evidence appraisal tools rather than as strong evidence to change routine clinical practice (Table 2).

## 3. Results

Nine RCTs were included in this review, published between 2016 and 2025. These trials assessed the analgesic efficacy of adjuncts added to local anaesthetic in ACB following TKA by comparing pain outcomes in groups that received an ACB with an adjunct vs. a group that received an ACB with local anaesthetic alone. Adjuncts included dexmedetomidine, dexamethasone, butorphanol, buprenorphine, and magnesium sulphate. The characteristics of the included RCTs are summarised in Table 3.

To improve clinical interpretability, we present findings by adjunct and primary outcome, while also highlighting key variables that differed between trials, summarised in Table 3. These contextual differences are likely to influence the observable impact of adjuncts, which limits direct comparison of absolute analgesic effects between studies.

### 3.1. Primary Outcomes

#### 3.1.1. Pain Scores (NRS or VAS) (*n* = 8)

Eight RCTs reported pain scores as a primary outcome using a visual analogue scale (VAS) or a numerical rating scale (NRS), as described in Table 4. Across three trials, dexmedetomidine 0.5 µg/kg improved pain scores compared with LA alone [17,19,21], whereas 0.25 µg/kg did not. These analgesic benefits were generally limited to the first 24 h and were not sustained at 48 h. In two trials, dexamethasone also showed early analgesic effects, particularly at doses ≥ 4 mg [20,22]. Wang et al. reported significantly lower pain scores at 6, 12, 18, and 24 h, while Turner et al. reported pain score improvements from 18–30 h. Similarly, these early analgesic benefits were not maintained at 48 h. A single study demonstrated reduced pain scores with butorphanol, with significant improvements in pain scores up to 24 h. However, again, no differences were observed between groups at 48 h [18]. Magnesium demonstrated inconsistent results across two trials, with Choi et al. demonstrating significantly lower pain scores, while Zoratto et al. did not.

#### 3.1.2. Time to First Rescue Analgesia (*n* = 5)

Five RCTs recorded the time to first rescue analgesia as a primary outcome, as described in Table 5. Across three trials, dexmedetomidine 0.5 μg/kg significantly prolonged the time to first analgesia compared to LA alone [17,19,21]. Lower doses (0.25 μg/kg) did not demonstrate a significant effect [21]. A single study highlighted that butorphanol prolonged the time to first PCIA press, though there was no significant difference in the rate of rescue analgesia between groups [18]. Magnesium sulphate did not significantly prolong the time to first rescue analgesia in Zoratto et al. [16], and this outcome was not reported in the other magnesium sulphate trial [15].

#### 3.1.3. Opioid Consumption (*n* = 9)

Nine RCTs collected data on total postoperative opioid consumption, as described in Table 6. Three RCTs reported significantly reduced postoperative opioid consumption with dexmedetomidine 0.5 μg/kg [17,19,21]. Again, lower doses (0.25 μg/kg) did not demonstrate this effect [21]. Dexamethasone showed variable effects on opioid consumption: an 8 mg dose significantly reduced 24-h morphine use in one trial, whereas 1–4 mg did not reduce 30-h opioid consumption in another, suggesting a possible dose–response that requires confirmation. Two RCTs assessing magnesium sulphate reported directly contradicting results on opioid consumption. Choi et al. reported significant reductions in oral morphine at both 24 h and 48 h [15]. In contrast, Zoratto et al. found no significant difference in opioid consumption over 24 h [16]. In a single study, butorphanol demonstrated significantly fewer PCIA attempts over 48 h, suggesting a reduction in opioid requirements [18]. Similarly, a single study revealed that buprenorphine significantly decreased postoperative opioid consumption compared to local anaesthetic alone [14].

#### 3.1.4. Sensory Block Duration (*n* = 3)

Objective assessment of sensory block duration was inconsistently measured across the studies, with only three RCTs measuring this objective outcome, shown in Table 7. Trials evaluating dexamethasone found that higher doses (4 mg and 8 mg) significantly extended the duration, while lower doses (1 mg) did not [20,22]. Butorphanol also produced significant prolongation of sensory block duration compared to the control group [18].

### 3.2. Secondary Outcomes

#### Functional Outcomes (*n* = 3)

Functional outcome measures were rarely reported, and definitions varied across trials, as illustrated in Table 8. Two RCTs found improvements in functional outcomes with dexmedetomidine. AbdelRady et al. found that the dexmedetomidine group had a significantly improved range of motion and performed better on the 100-foot test. Chattopadhyay et al. reported significantly greater quadriceps motor strength in the higher-dose dexmedetomidine group (*p* = 0.003), though improvements in steps walked did not reach statistical significance (*p* = 0.052) [17,21]. A single trial demonstrated that Butorphanol as an adjunct led to improvements in early knee range of motion, with no difference seen in mobilisation time, quadriceps strength, or Knee Society Score (KSS) function score [18].

### 3.3. Adverse Events

Adverse event reporting was incomplete in several studies, limiting firm conclusions about safety. However, across studies, dexmedetomidine, dexamethasone, butorphanol, magnesium and buprenorphine were generally well tolerated, with no increase in serious adverse events compared with local anaesthetic alone, as described in Table 9 and Table 10.

## 4. Discussion

Adequate analgesia following TKA is fundamental to improving rehabilitation and recovery. Several options have been studied, with regional anaesthesia being a favourable option in many institutions. The administration of adjuncts to local anaesthesia is an attractive and technically simple strategy to potentially extend the effects of peripheral nerve blocks beyond the conventional 8–14 h [24]. Many adjuncts have been explored for peripheral regional anaesthesia. However, their benefit in the context of single-shot ACBs following TKA is not yet conclusively established. To address this gap, we conducted a comprehensive systematic review of nine randomised controlled trials evaluating the analgesic effectiveness of adjuncts added to local anaesthetic in ACBs for TKA. Overall, evidence from the nine RCTs showed that perineural adjuncts to single-shot ACBs may provide meaningful analgesic benefits. However, no trial was judged to be at overall low risk of bias, and two were at high risk [14,19]. On GRADE assessment, certainty was generally moderate for dexmedetomidine and dexamethasone, but low or very low for most other adjuncts and outcomes. These ratings are based on a small number of trials with substantial clinical and methodological heterogeneity and no quantitative synthesis. Differences in local anaesthetic regimens, adjunct dosing, perioperative analgesic protocols, and outcome definitions likely contributed to variability in treatment effects and reduce comparability between studies. Accordingly, the certainty ratings in Table 2 should be viewed as supportive evidence appraisal tools that summarise the current limited evidence base, rather than as definitive clinical recommendations or standards of care. These agents should therefore be considered promising adjuncts, with any clinical use remaining cautious and context-specific pending further high-quality trials.

Despite this heterogeneity, dexmedetomidine and dexamethasone emerged as the most consistently effective adjuncts across the included trials. Dexmedetomidine demonstrated reproducible improvements in early postoperative analgesia across three RCTs at doses of 0.5 µg/kg, while lower doses (0.25 µg/kg) did not, suggesting a dose-dependent relationship, with a threshold dose of 0.5 µg/kg needed to produce significant effects [17,21]. Importantly, these analgesic effects were not sustained beyond 24 h, and by 48 h pain scores were similar between groups in the available trials. Dexmedetomidine was generally well tolerated. Transient and clinically insignificant early sedation was observed in one trial; however, no respiratory depression or haemodynamic instability was observed in either trial [17,21]. Overall, these findings suggest that dexmedetomidine may provide safe, dose-dependent improvements in early postoperative analgesia at doses of 0.5 µg/kg, but given that they are derived from a limited number of randomised trials, they should be regarded as preliminary and require confirmation in further studies.

Dexamethasone similarly decreased early postoperative pain following TKA and had the most evidence for significant prolongation in sensory blockade. A dose of ≥4 mg was needed to produce significant effects across trials. Wang et al. [20] revealed that the addition of dexamethasone (8 mg) to LA for ACB was able to prolong the duration of sensory block and decrease early postoperative pain, demonstrated by lower pain scores and reduced opioid consumption. Similarly, Turner et al. [22] reported that dexamethasone 4 mg, but not 1 mg, prolonged the duration of an ACB when measured by serial neurological pinprick exams [22], and improved pain scores. Despite this, no significant reduction in opioid consumption was observed in Turner et al. [22], even with prolonged sensory blockade. This may reflect the relatively long baseline block duration (~30 h in intervention and control group), suggesting that differences in analgesic outcomes may have become more apparent with longer follow-up. Although potential side effects of perineural dexamethasone were discussed as theoretical concerns in one trial [20], they were not formally reported in either. It is well established that systemic effects of dexamethasone may cause hyperglycaemia and increase the risk of postoperative infection, suggesting caution in use in patients with nerve damage and diabetes [25]. However, Turner et al. excluded all patients with diabetes mellitus, meaning the safety profile of perineural dexamethasone in this high-risk subgroup remains entirely uncharacterised [22]. Although the safety profile of dexamethasone as a perineural adjunct needs to be further characterised, no postoperative complications were reported across trials. Taken together, these findings support dexamethasone as a useful perineural adjunct in ACB, with the strongest evidence for prolonging sensory block duration, particularly at doses ≥4 mg. However, similar to dexmedetomidine, these conclusions are derived from a limited number of heterogeneous randomised trials and should be regarded as preliminary, warranting further confirmatory studies.

Evidence for buprenorphine and butorphanol as perineural adjuncts was limited to single RCTs, which limited the certainty of evidence and conclusions surrounding their analgesic efficacy. However, both adjuncts demonstrated promising results; buprenorphine added to LA demonstrated a significant reduction in postoperative opioid consumption, without increasing opioid-related adverse effects like nausea, vomiting, or pruritus [14]. Similarly, the addition of butorphanol to LA prolonged the duration of sensory block, relieved early postoperative pain, and improved the range of motion of the knee joint, without increasing the occurrence of postoperative adverse events or complications [18]. These improvements in pain outcomes, without a significant increase in side effects, make them promising anaesthetic adjuncts for TKA. However, repeated studies with further evidence are needed before their routine recommendation.

The evidence for magnesium sulphate as an adjunct to single-injection ACB was inconsistent across the two RCTs reviewed. Zoratto et al. [16] found that magnesium sulphate combined with ropivacaine did not significantly improve pain scores at any time point (2–48 h) or prolong time to rescue analgesia. Notably, this study incorporated neuraxial opioids and periarticular infiltration, which likely provided substantial baseline pain relief. In contrast, Choi et al. [15] reported that adding magnesium to bupivacaine significantly improved analgesic outcomes, with significantly lower pain scores at 24 and 48 h, and reduced opioid consumption over 48 h. The discrepancy in results suggests that the high baseline analgesia incorporated by Zoratto et al. may limit magnesium’s apparent efficacy as an adjunct. Magnesium was well tolerated, with no significant differences in nausea, vomiting or sedation reported across both trials. However, due to conflicting results and missing objective outcome measurements, further trials are required before its routine recommendation as an adjunct in ACB.

### 4.1. Clinical Implications

Based on the evidence synthesised from nine randomised controlled trials, dexmedetomidine and dexamethasone demonstrated the most consistent analgesic benefit among the adjuncts studied.

Dexmedetomidine at a dose of 0.5 μg/kg appears to provide the most reliable balance of early analgesic benefit and safety, whereas 0.25 μg/kg did not produce significant effects. Notably, although systemic α2-agonist effects such as sedation, bradycardia, and hypotension remain theoretical concerns, none of the included studies reported clinically significant haemodynamic adverse events at the 0.5 μg/kg dose. These effects may be dose-dependent and become more relevant at high systemic exposure. However, at the doses used across the included studies, systemic absorption appears negligible.

Dexamethasone at doses ≥ 4 mg appears to offer the greatest prolongation of sensory block duration and improvements in pain during mobilisation, whereas 1 mg provided limited benefit and did not meaningfully extend block duration. A 4 mg dose achieved the longest block duration observed in the review (37.9 ± 10.0 h) [22]. Although cross-study comparison is difficult due to clinical heterogeneity, this distinction is clinically relevant in the TKA population, where diabetes is common. Although 8 mg may offer additional benefit, further studies characterising doses and subsequent side effects are needed before it can be routinely recommended.

Buprenorphine (200 μg) and butorphanol (1 mg) show promise in reducing 24-h opioid requirements and extending early analgesia, though evidence remains limited to single-centre RCTs and broader validation is required before routine recommendation. Magnesium sulphate should be used with caution as a primary adjunct; its efficacy appears highly dependent on the baseline strength of the overall multimodal analgesic pathway.

### 4.2. Limitations

While the results generally favour the use of adjuncts, the studies in this review demonstrate substantial heterogeneity across multiple domains that are worth discussing.

#### 4.2.1. Local Anaesthetic Variability

LA agents varied between bupivacaine, levobupivacaine, and ropivacaine, each with distinct pharmacokinetic profiles that can affect the intensity and duration of a block. LA volumes also ranged from 10 to 30 mL, with consequent differences in area of neural coverage [26,27]. This means that studies utilising lower volumes or shorter-acting agents may report disproportionately large benefits from adjuncts compared to studies using high-volume, long-acting LAs where the baseline duration is already optimised.

#### 4.2.2. ACB Timing

ACB timing differed between preoperative (Wang et al., Turner et al.) and postoperative PACU administration (the majority). When performed before surgery, ACB may function as a form of pre-emptive analgesia, providing antinociceptive input prior to surgical incision [28]. By blocking sodium channels before tissue injury occurs, it may reduce the transmission of nociceptive signals to the spinal cord and help prevent the development of central sensitisation [28]. In contrast, postoperative ACB is administered after nociceptive input has already been established, at a point when the central nervous system may be in a hyperexcitable state. Some data suggest this distinction may be relevant, with one study reporting that preoperative ACB may reduce intraoperative opioid use and stabilise haemodynamics [29]. However, evidence for this remains limited and mixed. Taken together, the heterogeneity in ACB timing is an important consideration for cross-study comparison.

#### 4.2.3. The ‘Ceiling Effect’ of Multimodal Analgesia

Background anaesthetic techniques ranged from spinal alone to spinal plus intrathecal morphine, general anaesthesia, and varied systemic multimodal protocols. This represents a significant challenge in assessing adjunct efficacy. For instance, studies incorporating neuraxial opioids or comprehensive periarticular infiltration provide such substantial baseline pain relief that the incremental benefit of an ACB adjunct may be statistically masked. This likely explains the conflicting results seen with magnesium sulphate across different trial environments. Conversely, trials with less intensive background analgesia appeared more likely to demonstrate measurable benefit from adjuncts such as dexamethasone and dexmedetomidine.

#### 4.2.4. Outcome Measurement Gaps

Most current evidence is confined to the first 24 h post-surgery. Given that acute post-surgical TKA pain often peaks between 24 and 72 h [30] the clinical relevance of these adjuncts during the most critical recovery window remains under-investigated. Furthermore, the infrequent use of objective sensory testing (e.g., pinprick) means many studies rely on subjective pain scores, which are influenced by individual patient factors.

#### 4.2.5. Functional Outcomes

The majority of RCTs included in this study did not incorporate functional measures as outcomes. This represents a key limitation because the primary clinical rationale for adopting the ACB over the femoral nerve block is preservation of quadriceps motor function and facilitation of early ambulation. Functional recovery endpoints are arguably as important as analgesic duration or opioid consumption in this surgical context. Numerical pain scores are subjective and do not provide information on how pain affects recovery. This omission prevents any determination of whether adjuvant-mediated analgesic improvements translate into accelerated rehabilitation, earlier safe discharge, or meaningful patient-centred recovery.

## 5. Conclusions

Despite considerable clinical and methodological heterogeneity across the nine included RCTs, the available evidence suggests that pharmacological adjuncts added to single-shot ACBs can improve early postoperative analgesia following TKA compared with LA alone. Across multiple trials, dexmedetomidine 0.5 µg/kg and dexamethasone at doses ≥ 4 mg provide the most consistent benefits in terms of lower early pain scores, reduced 24-h opioid consumption, and prolonged sensory block duration, although these effects are largely confined to the first 24 h after surgery.

Adjuncts such as butorphanol and buprenorphine may also reduce opioid requirements and improve early pain outcomes, but supporting evidence is currently restricted to single, small RCTs, and findings for magnesium sulphate remain conflicting. Overall, the certainty of evidence is moderate to low due to risk of bias, imprecision, and inconsistent outcome measurement. Functional recovery outcomes and longer-term pain trajectories are rarely reported. Taken together with the small number of studies, substantial heterogeneity, and the absence of quantitative synthesis, the certainty ratings presented should be interpreted as an evidence-appraisal framework rather than as prescriptive clinical recommendations, and these agents should be regarded as promising adjuncts rather than established standards of care in ACB-based TKA analgesia.

Future research should focus on well-designed, adequately powered RCTs with harmonised definitions of pain, opioid consumption, and functional recovery, and with follow-up extending beyond the initial 24-h window. Such studies are needed to determine the optimal dosing and combination of adjuncts, to better characterise safety, and to clarify whether improvements in early analgesia translate into clinically meaningful gains in mobilisation, rehabilitation, and patient-centred outcomes after TKA.

## Figures and Tables

**Table 1:** Cochrane Risk of Bias 2 (RoB 2) assessment for included randomised controlled trials.

Study	D1: Randomisation Process	D2: Deviations from Intended Interventions	D3: Missing Outcome Data	D4: Outcome Measurement	D5: Selection of Reported Result	Overall RoB
2016 Krishnan et al. [14]	Some Concerns	Some Concerns	Some Concerns	Low	High	High
2022 Choi et al. [15]	Some Concerns	Some Concerns	Some Concerns	Low	Low	Some Concerns
2021 Zoratto et al. [16]	Low	Some Concerns	Some Concerns	Low	Low	Some Concerns
2021 AbdelRady et al. [17]	Some Concerns	Some Concerns	Low	Low	Low	Some Concerns
2022 Mu et al. [18]	Low	Low	Some Concerns	Low	Low	Some Concerns
2025 Rohan et al. [19]	Low	Low	Low	Low	High	High
2017 Wang et al. [20]	Low	Some Concerns	Low	Low	Low	Some Concerns
2022 Chattopadhyay et al. [21]	Some Concerns	Some Concerns	Low	Low	Low	Some Concerns
2018 Turner et al. [22]	Low	Low	Some Concerns	Low	Some Concerns	Some Concerns

RoB, risk of bias; D, domain. D1: randomisation process; D2: deviations from intended interventions; D3: missing outcome data; D4: measurement of the outcome; D5: selection of the reported result. RoB 2 categories: “Low” = low risk of bias; “Some Concerns” = some concerns; “High” = high risk of bias.

**Table 2:** Narrative GRADE overview of pharmacological adjuncts to single-shot adductor canal block in total knee arthroplasty (small, heterogeneous RCTs; no quantitative pooling).

Adjunct	Outcome	No. of Studies (Participants)	Risk of Bias	Inconsistency	Indirectness	Imprecision	Publication Bias	Certainty
Dexmedetomidine 0.5 µg/kg	Pain scores (NRS/VAS)	3 RCTs [17,19,21] (*n* = 255)	Serious risk of bias (−1)	No serious inconsistency	Not serious	Not serious	Undetected	⊕⊕⊕◯ MODERATE
Dexmedetomidine 0.5 µg/kg	Time to first rescue analgesia	3 RCTs [17,19,21] (*n* = 255)	Serious risk of bias (−1)	No serious inconsistency	Not serious	Not serious	Undetected	⊕⊕⊕◯ MODERATE
Dexmedetomidine 0.5 µg/kg	Opioid consumption	3 RCTs [17,19,21] (*n* = 255)	Serious risk of bias (−1)	No serious inconsistency	Not serious	Not serious	Undetected	⊕⊕⊕◯ MODERATE
Dexamethasone (1 mg, 4 mg, 8 mg)	Pain scores (NRS/VAS)	2 RCTs [20,22] (*n* = 271)	Serious risk of bias (−1)	No serious inconsistency	Not serious	Not serious	Undetected	⊕⊕⊕◯ MODERATE
Dexamethasone (1 mg, 4 mg, 8 mg)	Opioid consumption	2 RCTs [20,22] (*n* = 271)	Serious risk of bias (−1)	Serious inconsistency (−1)	Not serious	Not serious	Undetected	⊕⊕◯◯ LOW
Dexamethasone (1 mg, 4 mg, 8 mg)	Sensory block duration	2 RCTs [20,22] (*n* = 271)	Serious risk of bias (−1)	No serious inconsistency	Not serious	Not serious	Undetected	⊕⊕⊕◯ MODERATE
Butorphanol 1 mg	Pain scores (NRS/VAS)	1 RCT [18] (*n* = 70)	Serious risk of bias (−1)	Not applicable (single study)	Not serious	Serious (−1)	Undetected	⊕⊕◯◯ LOW
Butorphanol 1 mg	Time to first rescue analgesia (time to first PCIA press)	1 RCT [18] (*n* = 70)	Serious risk of bias (−1)	Not applicable (single study)	Not serious	Serious (−1)	Undetected	⊕⊕◯◯ LOW
Butorphanol 1 mg	Opioid consumption (PCIA attempts)	1 RCT [18] (*n* = 70)	Serious risk of bias (−1)	Not applicable (single study)	Serious (−1)	Serious (−1)	Undetected	⊕◯◯◯ VERY LOW
Butorphanol 1 mg	Sensory block duration	1 RCT [18] (*n* = 70)	Serious risk of bias (−1)	Not applicable (single study)	Not serious	Serious (−1)	Undetected	⊕⊕◯◯ LOW
Magnesium sulphate	Pain scores (NRS/VAS)	2 RCTs [15,16] (*n* = 240)	Serious risk of bias (−1)	Very serious (−2)	Not serious	Serious (−1)	Undetected	⊕◯◯◯ VERY LOW
Magnesium sulphate	Opioid consumption	2 RCTs [15,16] (*n* = 240)	Serious risk of bias (−1)	Very serious (−2)	Not serious	Serious (−1)	Undetected	⊕◯◯◯ VERY LOW
Buprenorphine 200 µg	Opioid consumption (hydrocodone equiv.)	1 RCT [14] (*n* = 100)	Very serious risk of bias (−2)	Not applicable (single study)	Not serious	Serious (−1)	Undetected	⊕◯◯◯ VERY LOW

Note: Certainty ratings are derived from a small number of heterogeneous RCTs without quantitative synthesis (no meta-analysis). They are intended solely as supportive evidence appraisal tools to help interpret the current limited literature and should not be viewed as definitive estimates of treatment effect or as clinical practice recommendations. GRADE, Grading of Recommendations Assessment, Development and Evaluation. Certainty ratings: ⊕⊕⊕⊕ = high, ⊕⊕⊕◯ = moderate, ⊕⊕◯◯ = low, ⊕◯◯◯ = very low. Downgrading reasons (−1, −2) are shown in each domain; RCT, randomised controlled trial; VAS, visual analogue scale; NRS, numerical rating scale; PCIA, patient-controlled intravenous analgesia.

**Table 3:** Characteristics of included randomised controlled trials.

Study	Design	*n*	Adjunct (Dose)	Local Anaesthetic	Anaesthetic and Analgesic Technique (Neuraxial ± PAI)	Comparator	ACB Timing	Primary Outcomes Reported	Overall RoB
2021 AbdelRady et al. [17]	RCT, double-blind	60	Dexmedetomidine 0.5 µg/kg	20 mL levobupivacaine 0.25%	Spinal Neuraxial Opioid: No PAI: No	Single-shot ACB + saline	Post-operative	Pain scores (VAS), time to first rescue analgesia, opioid consumption, sensory block duration	Some concerns
2022 Chattopadhyay et al. [21]	RCT, double-blind, 3-arm	135	Dexmedetomidine 0.25 µg/kg or 0.5 µg/kg	20 mL ropivacaine 0.375%	Spinal +/− GA Neuraxial opioid: No PAI: No	Single-shot ACB + saline	Post-operative	Pain scores (NRS), time to first rescue analgesia, opioid consumption, functional outcomes (Barthel Index)	Some concerns
2025 Rohan et al. [19]	RCT, double-blind	60	Dexmedetomidine 0.5 µg/kg	20 mL levobupivacaine 0.25%	Spinal Neuraxial Opioid: Intrathecal fentanyl PAI: No	Single-shot ACB + saline	Post-operative	Pain scores (NRS), time to first rescue analgesia, opioid consumption	High
2018 Turner et al. [22]	RCT, double-blind, 3-arm	85	Dexamethasone 1 mg or 4 mg	20 mL ropivacaine 0.5%	Spinal +/− GA Neuraxial Opioid: Intrathecal fentanyl PAI: No	Single-shot ACB + saline (placebo)	Pre-operative	Pain scores (NRS), time to first rescue analgesia, opioid consumption, sensory block duration	Some concerns
2017 Wang et al. [20]	RCT, double-blind	186	Dexamethasone 8 mg	20 mL ropivacaine 0.33%	Spinal Neuraxial Opioid: No PAI: No	Single-shot ACB + saline	Pre-operative	Pain scores (VAS), time to first rescue analgesia, opioid consumption, sensory block duration	Some concerns
2022 Mu et al. [18]	RCT, double-blind	70	Butorphanol 1 mg	20 mL ropivacaine 0.33%	GA Neuraxial Opioid: No PAI: No	Single-shot ACB + saline	Post-operative	Pain scores (VAS), time to first PCIA press, opioid consumption (PCIA attempts), sensory block duration	Some concerns
2021 Zoratto et al. [16]	RCT, double-blind	121	Magnesium sulphate 2 g	10 mL ropivacaine 0.5%	Spinal Neuraxial Opioid: Intrathecal morphine PAI: Yes	Single-shot ACB + saline	Post-operative	Pain scores (VAS), time to first rescue analgesia, opioid consumption	Some concerns
2022 Choi et al. [15]	RCT, double-blind	119	Magnesium sulphate 150 mg	30 mL bupivacaine 0.25%	Spinal Neuraxial Opioid: No PAI: Yes	Single-shot ACB + saline	Post-operative	Pain scores (VAS), opioid consumption, functional outcomes (steps walked, ROM)	Some concerns
2016 Krishnan et al. [14]	RCT, double-blind	100	Buprenorphine 200 µg	20 mL bupivacaine 0.25%	Spinal Neuraxial Opioid: No PAI: No	Single-shot ACB + saline	Post-operative	Opioid consumption (hydrocodone equivalents), adverse events	High

ACB, adductor canal block; PAI, periarticular infiltration; GA; general anaesthetic; PCIA, patient-controlled intravenous analgesia; NRS, numerical rating scale; VAS, visual analogue scale; ROM, range of motion; RoB, risk of bias.

**Table 4:** Patient-reported pain scores (VAS or NRS) in studies comparing single-shot ACBs with and without adjuncts following TKA.

Study	Local Anaesthetic	Adjuvant (Dose)	Pain Scale	4–12 h	24 h	48 h	Summary of Significant Findings
2022 Mu et al. [18]	20 mL 0.33% ropivacaine	1 mg butorphanol	VAS	Significantly lower VAS at 4 h, 8 h, 12 h at rest and with activity (*p* < 0.01)	Significantly lower at 24 h at rest (*p* < 0.01); no significant difference at 24 h with activity	No significant difference at 48 h (rest or movement)	Butorphanol group had lower pain scores at rest within 24 h and within 12 h with activity; differences diminished by 48 h.
2021 Zoratto et al. [16]	10 mL 0.5% ropivacaine	2 g MgSO_4_	VAS	No significant difference at any time point	No significant difference	No significant difference	No significant differences in VAS at 2, 4, 8, 12, 18, 24, or 48 h between magnesium and control groups.
2022 Choi et al. [15]	30 mL 0.25% bupivacaine	150 mg MgSO_4_	VAS	Not reported	Significantly lower VAS at 24 h in magnesium group (*p* < 0.001)	Significantly lower VAS at 48 h in magnesium group (*p* < 0.001)	Magnesium group had significantly lower pain scores at 24 h and 48 h compared with control.
2021 AbdelRady et al. [17]	20 mL 0.25% levobupivacaine	Dexmedetomidine 0.5 µg/kg	VAS	Significantly lower scores at 6 h, 8 h, and 12 h (*p* < 0.05)	No significant difference at 24 h at rest; significantly lower VAS at 24 h with mobilisation (*p* < 0.05)	No significant difference at 48 h at rest	Dexmedetomidine group had lower pain scores up to 24 h (especially with mobilisation); differences were not maintained at 48 h.
2025 Rohan et al. [19]	20 mL 0.25% levobupivacaine	Dexmedetomidine 0.5 µg/kg	NRS	Significantly lower NRS at 2–10 h (*p* < 0.05); not significant at 12 h	Not reported	Not reported	Dexmedetomidine significantly improved early postoperative analgesia in the first 10 h; no sustained difference by 12 h.
2022 Chattopadhyay et al. [21]	20 mL 0.375% ropivacaine	Dexmedetomidine 0.25 µg/kg	NRS	No significant difference at any time (rest or movement)	No significant difference	Not reported	0.25 µg/kg dexmedetomidine did not significantly change pain scores at any time point.
2022 Chattopadhyay et al. [21]	20 mL 0.375% ropivacaine	Dexmedetomidine 0.5 µg/kg	NRS	Significantly lower NRS at 12 h with movement (*p* < 0.05)	Significantly lower at 18 h and 24 h at rest and with movement (*p* < 0.05)	Not reported	0.5 µg/kg dexmedetomidine reduced pain scores at 12–24 h, with significant differences at 18–24 h at rest and at 12, 18, and 24 h with movement.
2018 Turner et al. [22]	20 mL 0.25% bupivacaine	Dexamethasone 1 mg	NRS	No significant difference at any time, at rest or with movement	Significant reduction in NRS at 18 h and 30 h with movement only (*p* < 0.05)	Not reported	No improvement in pain scores at rest at any time; minor improvements at 18 and 30 h with movement only.
2018 Turner et al. [22]	20 mL 0.25% bupivacaine	Dexamethasone 4 mg	NRS	No significant difference at early time points with rest or movement	Significant reduction at 18 h and 24 h at rest (*p* < 0.05); significant reduction at 18, 24, and 30 h with movement (*p* < 0.05)	Not reported	Multiple significant differences from 18 h onwards, especially with movement; no between-group differences before 18 h.
2017 Wang et al. [20]	10 mL 0.5% ropivacaine	Dexamethasone 8 mg	NRS	Lower scores at 6 h and 12 h (*p* < 0.001) at rest and with movement	Lower scores at 18 h and 24 h (*p* < 0.001) at rest and with movement	No significant difference at 48 h (rest or movement)	Dexamethasone 8 mg provided clear analgesic benefit from 6–24 h, which was not maintained at 48 h.

VAS, visual analogue scale; NRS, numerical rating scale. “Significantly lower” refers to between-group comparisons (adjunct vs. control) as reported in the original studies.

**Table 5:** Time to first rescue analgesia in studies comparing single-shot ACBs with vs. without adjuncts following TKA.

Study	Local Anaesthetic	Adjuvant (Dose)	Time to First Rescue Analgesia—Intervention	Time to First Rescue Analgesia—Control	Significant Difference
2021 Zoratto et al. [16]	10 mL 0.5% ropivacaine	2 g of 10% MgSO_4_	270 min [113–780]	298 min [120–776]	No (*p* = 0.96)
2022 Mu et al. [18]	20 mL 0.33% ropivacaine	1 mg butorphanol	20.31 ± 2.59 h	16.25 ± 2.31 h	Yes (*p* < 0.01)
2021 AbdelRady et al. [17]	20 mL 0.25% levobupivacaine	Dexmedetomidine 0.5 µg/kg	515.10 ± 27.98 min	406.77 ± 10.64 min	Yes (*p* < 0.001)
2022 Chattopadhyay et al. [21]	20 mL 0.375% ropivacaine	Dexmedetomidine 0.25 µg/kg	16.44 ± 6.21 h	15.71 ± 4.87 h	No
2022 Chattopadhyay et al. [21]	20 mL 0.375% ropivacaine	Dexmedetomidine 0.5 µg/kg	19.78 ± 5.57 h	15.71 ± 4.87 h	Yes (*p* = 0.014)
2025 Rohan et al. [19]	20 mL 0.25% levobupivacaine	Dexmedetomidine 0.5 µg/kg	7.7 (6.0–9.0) h	6.3 (5.0–7.5) h	Yes (*p* = 0.036)

Time to first rescue analgesia is reported using the original study definitions. In Mu et al., this outcome reflects the time to first PCIA button press rather than protocol-defined rescue analgesia. Units (minutes vs. hours) and summary measures (mean ± SD vs. median [IQR] or range) vary across studies and should not be directly compared quantitatively.

**Table 6:** Postoperative opioid consumption at reported timepoints in studies comparing single-shot ACBs with and without adjuncts following TKA.

Study	Local Anaesthetic	Adjuvant (Dose)	Timepoints Assessed	Intervention Group Opioid Use	Control Group Opioid Use	Significant Difference
2016 Krishnan et al. [14]	0.25% bupivacaine	Buprenorphine 200 µg	24 h	25.3 mg OME	35.8 mg OME	Yes (*p* = 0.0076)
2021 Zoratto et al. [16]	10 mL 0.5% ropivacaine	2 g of 10% MgSO_4_	24 h	No observed difference in total opioid consumption	No observed difference in total opioid consumption	No (*p* = 0.94)
2022 Choi et al. [15]	30 mL 0.25% bupivacaine	150 mg MgSO_4_	24 h	21.3 ± 2.4 mg OME (24 h)	33.2 ± 3.0 mg OME (24 h)	Yes (*p* = 0.003)
			48 h	27.3 ± 2.3 mg oral morphine equivalents (48 h)	35.4 ± 2.7 mg (48 h)	Yes (*p* = 0.026)
2021 AbdelRady et al. [17]	20 mL 0.25% levobupivacaine	Dexmedetomidine 0.5 µg/kg	24 h	6.47 mg OME	10.93 mg OME	Yes (*p* < 0.05)
2022 Chattopadhyay et al. [21]	20 mL 0.375% ropivacaine	Dexmedetomidine 0.25 µg/kg	24 h	5.22 ± 1.9 mg OME	6.0 ± 1.4 mg OME	Not significant
2022 Chattopadhyay et al. [21]	20 mL 0.375% ropivacaine	Dexmedetomidine 0.5 µg/kg	24 h	3.33 ± 1.40 mg OME	6.0 ± 1.39 mg OME	Yes (*p* = 0.033)
2025 Rohan et al. [19]	20 mL 0.25% levobupivacaine	Dexmedetomidine 0.5 µg/kg	24 h	10 ± 4 mg OME	14 ± 5 mg OME	Yes (*p* = 0.002)
2018 Turner et al. [22]	20 mL 0.25% bupivacaine	Dexamethasone 1 mg	30 h cumulative	39.0 mg ± 37.2 mg OME	63.5 mg ± 39.5 mg OME	Not significant
2018 Turner et al. [22]	20 mL 0.25% bupivacaine	Dexamethasone 4 mg	30 h cumulative	39.6 mg ± 36.15 mg OME	63.5 mg ± 39.5 mg OME	Not significant
2017 Wang et al. [20]	10 mL 0.5% ropivacaine	Dexamethasone 8 mg	24 h	4.23 ± 1.80 mg OME	8.42 ± 2.44 mg OME	Yes (*p* < 0.05)
2022 Mu et al. [18]	20 mL 0.33% ropivacaine	Butorphanol 1 mg	48 h	Fewer PCIA attempts (4.5 ± 1.2)	More PCIA attempts (7.8 ± 1.5)	Yes (*p* < 0.05)

Opioid doses were converted to oral morphine equivalents (OME) using standard equianalgesic ratios (e.g., morphine 1:1, oxycodone 1:1.5, hydrocodone 1:1, tramadol 10:1). Where conversion was not feasible (e.g., PCIA attempts only), original measures were retained and noted.

**Table 7:** Objective sensory block duration in studies comparing single-shot ACBs with and without adjuncts following TKA.

Study	Local Anaesthetic	Adjuvant (Dose)	Method of Sensory Assessment	Intervention Group Duration	Control Group Duration	Significant Difference
2022 Mu et al. [18]	20 mL 0.33% ropivacaine	Butorphanol 1 mg	Sensory block assessment (clinical)	18.42 ± 3.46 h	15.36 ± 2.29 h	Yes (*p* < 0.01)
2017 Wang et al. [20]	10 mL 0.5% ropivacaine	Dexamethasone 8 mg	Serial pinprick testing	23.42 ± 3.35 h	14.67 ± 2.96 h	Yes (*p* < 0.05)
2018 Turner et al. [22]	20 mL 0.25% bupivacaine	Dexamethasone 1 mg	Serial pinprick examinations	31.8 ± 10.5 h	29.7 ± 6.8 h	No
2018 Turner et al. [22]	20 mL 0.25% bupivacaine	Dexamethasone 4 mg	Serial pinprick examinations	37.9 ± 10.0 h	29.7 ± 6.8 h	Yes (*p* = 0.011)

Sensory block duration was assessed using pinprick or similar modalities as reported in the original studies and is presented as mean ± SD (hours). SD, standard deviation.

**Table 8:** Postoperative functional outcomes in studies comparing single-shot ACBs with and without adjuncts following TKA.

Study	LA	Adjuvant	Functional Outcomes Assessed	Main Findings
2021 AbdelRady et al. [17]	Levobupivacaine 0.25%	Dexmedetomidine 0.5 µg/kg	Knee range of motion (ROM); 100-foot walking test	Dexmedetomidine group had significantly improved ROM and better 100-foot walking test performance (*p* = 0.003).
2022 Chattopadhyay et al. [21]	Ropivacaine 0.375%	Dexmedetomidine 0.25 µg/kg and 0.5 µg/kg	Motor strength (straight-leg raise/MRC); ambulation steps	Higher-dose dexmedetomidine (0.5 µg/kg) was associated with better ambulation and motor strength than lower dose and control.
2022 Mu et al. [18]	Ropivacaine 0.33%	Butorphanol 1 mg	Knee ROM; quadriceps strength; time to first mobilisation; Knee Society Score (KSS) function	Butorphanol improved early knee ROM; no significant differences in time to first mobilisation, quadriceps strength, or KSS function score.

ROM, range of motion; KSS, Knee Society Score; MRC, Medical Research Council muscle strength scale (0–5, higher scores indicate greater muscle power).

**Table 9:** Adverse events reported across studies comparing single-shot ACBs with and without adjuncts following TKA.

Study	Adjunct	Adverse Events Monitored	Adverse Events Reported	Conclusion
2016 Krishnan et al. [14]	Buprenorphine	Nausea, vomiting, pruritus	No significant differences between groups	Buprenorphine did not increase opioid-related side effects.
2022 Choi et al. [15]	Magnesium sulphate	Nausea/vomiting (48 h)	No significant difference between groups	Magnesium did not increase nausea or vomiting.
2021 Zoratto et al. [16]	Magnesium sulphate	Nausea, pruritus, sedation (24 h)	No significant difference between groups	No differences between groups in any monitored adverse event.
2021 AbdelRady et al. [17]	Dexmedetomidine 0.5 µg/kg	Sedation, neurological deficit, bleeding, infection	No serious complications reported	No major adverse effects observed in either group.
2022 Mu et al. [18]	Butorphanol 1 mg	PONV, pruritus, constipation, somnolence, urinary retention, respiratory depression	No significant difference between groups	No significant differences in any recorded complication.
2025 Rohan et al. [19]	Dexmedetomidine 0.5 µg/kg	Hypotension, bradycardia, nausea/vomiting, pruritus, sedation, neurological deficit	No events recorded	No clinically significant adverse effects reported.
2017 Wang et al. [20]	Dexamethasone 8 mg	Not formally prespecified	No formal adverse-event data reported	Adverse-event reporting insufficient; potential neurotoxicity discussed only in text.
2022 Chattopadhyay et al. [21]	Dexmedetomidine 0.25–0.5 µg/kg	Shivering, giddiness, nausea, vomiting, paraesthesia, haemodynamics	No adverse events reported in any patient across all three groups	No adverse effects observed in any group.
2018 Turner et al. [22]	Dexamethasone 1 mg vs. 4 mg	Not formally prespecified; diabetics excluded	No formal adverse-event outcomes reported; diabetic patients excluded	Reporting insufficient; safety not generalisable to diabetic patients.

PONV, post-operative nausea/vomiting.

**Table 10:** Summary of findings for pharmacological adjuncts to single-shot adductor canal block in total knee arthroplasty.

Adjunct (Dose)	Main Analgesic Effect vs. LA Alone	Functional Outcomes	Certainty of Evidence (Overall)	Key Limitations/Comments
Dexmedetomidine 0.5 µg/kg	Consistently reduced early postoperative pain scores (up to 24 h) and prolonged time to first rescue analgesia; reduced 24 h opioid consumption across three RCTs. Effect not maintained at 48 h.	Two trials reported improved early functional markers (ROM, quadriceps strength, 100-foot test), but measures were heterogeneous and follow-up short.	Moderate for pain, time to first rescue, and opioid consumption; low for functional outcomes.	No trial at low risk of bias; some high risk due to registration/Selective reporting. Heterogeneity in LA type and volume, ACB timing, and background multimodal pathways. Follow-up generally limited to 24–48 h and functional outcomes sparsely reported.
Dexmedetomidine 0.25 µg/kg	Did not consistently improve pain scores or reduce opioid consumption compared with LA alone in the single RCT that evaluated this dose.	No clear functional benefit demonstrated.	Low.	Evidence limited to one trial; moderate risk of bias. Dose–response inferences remain tentative.
Dexamethasone 4–8 mg	Two RCTs showed improved early pain scores and reduced opioid consumption with higher doses (particularly 8 mg); lower dose (1 mg) had little or no effect on pain or opioids. Sensory block duration significantly prolonged at 4–8 mg.	No consistent functional benefit demonstrated; functional outcomes infrequently and variably reported.	Moderate for pain and sensory duration; low for opioid outcomes and functional outcomes.	Only two RCTs with differing doses and regimens; some concerns for risk of bias. Dosing range is narrow and follow-up limited to 24–48 h. Dose–response observations should be interpreted cautiously and require confirmation.
Butorphanol 1 mg	Single RCT showed lower pain scores in the first 24 h and delayed time to first PCIA activation, with fewer PCIA attempts over 48 h, suggesting an opioid-sparing effect. No difference at 48 h pain scores.	One trial reported improved early knee range of motion, with no clear benefits in mobilisation time, quadriceps strength, or composite functional scores.	Low to very low.	Evidence from one moderate–high risk-of-bias RCT only. Outcome definitions (PCIA attempts) and background analgesia limit generalisability. No data beyond early postoperative period.
Buprenorphine 200 µg	Single RCT reported reduced 24 h opioid consumption (hydrocodone-equivalents) with no major safety concerns. Pain scores were not consistently reported as a primary outcome.	Functional outcomes were not reported.	Very low.	Very serious risk of bias and reliance on a single study; limited outcome set. Lack of functional data and short follow-up preclude firm clinical recommendations.
Magnesium sulphate (150 mg–2 g)	Analgesic findings inconsistent: one RCT showed reduced pain scores and opioid consumption at 24–48 h, whereas another found no difference in pain or opioids.	One trial measured steps walked and ROM, with no clear improvement attributable to magnesium.	Very low for both pain and opioid outcomes.	Marked inconsistency between the two RCTs, differences in dose, LA regimen, and background analgesia. High overall risk of bias and imprecision; any apparent benefit remains highly uncertain.

ROM, range of motion; RCT, randomised controlled trial; LA, local anaesthetic; PCIA, patient-controlled analgesia; ACB, adductor canal block.

## Data Availability

The original contributions presented in this study are included in the article/Supplementary Materials. Further inquiries can be directed to the corresponding author.

## Supplementary Materials

The following supporting information can be downloaded at: https://www.mdpi.com/article/10.3390/jcm15135005/s1, PRISMA 2020 Checkilist; Methods—Search Strategies. Reference [31] is cited in Supplementary Materials.
