# Supplementary material for: Extending the Window: A Systematic Review of Pharmacological Adjuncts for Single-Shot Adductor Canal Blocks in Total Knee Arthroplasty
Source: J Clin Med. 2026 Jun 26;15(13):5005. doi: 10.3390/jcm15135005 (PMC13363162; doi:10.3390/jcm15135005)
Supplement: Supplementary file 1 [file jcm-15-05005-s001.zip › Methods - Search Strategies.pdf]

4760 Search Strategies 19 February 2026

Medline (Ovid)

1 Arthroplasty, Replacement, Knee/ (37223)
2 (knee adj2 (replacement or arthroplasty)).tw. (47611)
3 TKA.tw. (21195)
4 or/1-3 (55003)
5 ((adductor canal or saphenous nerve or subsartorial or hunter*) adj3 block*).tw. (870)
6 anesthesia, conduction/ or nerve block/ (31833)
7 ((regional or conduction) adj2 (anaesthes* or anesthes*)).tw. (12495)
8 ((nerve or conduction) adj2 block*).tw. (24307)
9 nerve infiltration.tw. (408)
10 or/6-9 (52626)
11 (adductor canal or saphenous nerve or subsartorial or hunter*).tw. (13369)
12 10 and 11 (819)
13 acb.tw. (5549)
14 or/5,12-13 (6190)
15 4 and 14 (585)
16 (adjunct* or adjuvant* or additive*).tw. (502625)
17 Dexamethasone/ or Dexmedetomidine/ or Clonidine/ or Buprenorphine/ or Epinephrine/ (142673)
18 (dexamethasone or dexmedetomidine or clonidine or buprenorphine or epinephrine).tw,nm. (201218)
19 perineural.tw. (11532)
20 or/16-19 (707598)
21 15 and 20 (78)

Embase (Elsevier)

No.	Query	Results
1	'knee arthroplasty'/de OR 'knee replacement'/de	30568
2	(knee NEAR/1 (replacement OR arthroplasty)):ti,ab	59124
3	tka:ti,ab	24779
4	#1 OR #2 OR #3	70435
5	'saphenous nerve'/de	2423
6	'adductor canal':ti,ab OR 'saphenous nerve':ti,ab OR subsartorial:ti,ab OR hunter*:ti,ab	17224
7	#5 OR #6	18110
8	'nerve block'/de	51890
9	((nerve OR conduction) NEAR/1 block*):ti,ab	37016
10	'regional anesthesia'/de	31418
11	(regional NEAR/1 (anaesthes* OR anesthes*)):ti,ab	21975
12	'nerve infiltration':ti,ab	568

13	#8 OR #9 OR #10 OR #11 OR #12	90625
14	#7 AND #13	2030
15	acb:ti,ab OR ((('adductor canal' OR 'saphenous nerve' OR subsartorial OR hunter*) NEAR/2 block*):ti,ab)	4318
16	#14 OR #15	4883
17	#4 AND #16	1150
18	'adjuvant'/de	50978
19	adjuvant*:ti,ab OR adjunct*:ti,ab OR additive*:ti,ab	694667
20	'dexamethasone'/de OR 'dexmedetomidine'/de OR 'clonidine'/de OR 'buprenorphine'/de OR 'epinephrine'/de	471521
21	dexamethasone:ti,ab OR dexmedetomidine:ti,ab OR clonidine:ti,ab OR buprenorphine:ti,ab OR epinephrine:ti,ab OR perineural:ti,ab	237293
22	#18 OR #19 OR #20 OR #21	1195750
23	#17 AND #22	396
24	#17 AND #22 AND ([embase]/lim OR [medline]/lim OR [preprint]/lim OR [pubmed-not-medline]/lim)	297
25	#24 AND 'Conference Abstract'/it	80
26	#24 NOT #25	217

CINAHL (Ebsco)

Search ID#	Search Terms	Results
S1	(MH "Arthroplasty, Replacement, Knee")	20,920
S2	XB (knee N1 (replacement or arthroplasty)	23,130
S3	XB TKA	8,307
S4	S1 OR S2 OR S3	28,520
S5	XB (("adductor canal" or "saphenous nerve" or subsartorial or hunter*) N2 block*)	445
S6	(MH "Anesthesia, Conduction") OR (MH "Nerve Block")	13,203
S7	XB ((regional or conduction) N1 (anaesthes* or anesthes*))	3,684
S8	XB ((nerve or conduction) N1 block*)	7,237
S9	XB "nerve infiltration"	67
S10	S6 OR S7 OR S8 OR S9	17,989
S11	XB ("adductor canal" or "saphenous nerve" or subsartorial or hunter*)	2,370
S12	S10 AND S11	407
S13	XB "acb"	412
S14	S5 OR S12 OR S13	768

S15	S4 AND S14	282
S16	XB (adjunct* or adjuvant* or additive*)	75,920
S17	(MH "Dexamethasone")	7,326
S18	(MH "Clonidine")	1,626
S19	(MH "Buprenorphine")	5,299
S20	(MH "Epinephrine")	6,271
S21	XB (dexamethasone or dexmedetomidine or clonidine or buprenorphine or epinephrine or prineural)	23,617
S22	S16 OR S17 OR S18 OR S19 OR S20 OR S21	106,684
S23	S15 AND S22	21

Cochrane CENTRAL database of Controlled Trials, Issue 1, January 2026

ID Search
#1 MeSH descriptor: [Arthroplasty, Replacement, Knee] this term only
#2 (knee NEAR/2 (arthroplasty or replacement*)):ti,ab,kw
#3 (TKA):ti,ab,kw
#4 #1 OR #2 OR #3
#5 (("adductor canal" or "saphenous nerve" or subsartorial or hunter*) NEAR/2 block*):ti,ab,kw
#6 MeSH descriptor: [Anesthesia, Conduction] this term only
#7 MeSH descriptor: [Nerve Block] this term only
#8 ((regional or conduction) NEAR/2 (anaesthes* or anesthes*)):ti,ab,kw
#9 ((nerve or conduction) NEAR/2 block*):ti,ab,kw
#10 ("nerve infiltration"):ti,ab,kw
#11 #6 OR #7 OR #8 OR #9 OR #10
#12 ("adductor canal" OR "saphenous nerve" OR subsartorial or hunter*):ti,ab,kw
#13 #11 AND #12
#14 ("ACB"):ti,ab,kw
#15 #5 OR #13 OR #14
#16 #4 AND #15
#17 (adjunct* or adjuvant* or additive*):ti,ab,kw
#18 MeSH descriptor: [Dexamethasone] this term only
#19 MeSH descriptor: [Dexmedetomidine] this term only
#20 MeSH descriptor: [Clonidine] this term only
#21 MeSH descriptor: [Buprenorphine] this term only
#22 MeSH descriptor: [Epinephrine] this term only
#23 (dexamethasone or dexmedetomidine or clonidine or buprenorphine or epinephrine):ti,ab,kw
#24 (perineural):ti,ab,kw
#25 {OR #17-#24}
#26 #16 AND #25

Web of Science (Clarivate)

Search: #8 AND #7
Results: 127

Search: adjunct* or adjuvant* or additive* or perineural or dexamethasone or dexmedetomidine or clonidine or buprenorphine or epinephrine (Topic)
Results: 1160211

Search: #1 AND #6
Results: 794

Search: #4 OR #5
Results: 4090

Search: ("adductor canal" or "saphenous nerve" or subsartorial or hunter*) NEAR/2 block* (Topic) OR ACB (Topic)
Results: 3950

Search: #2 AND #3
Results: 1037

Search: "adductor canal" OR "saphenous nerve" OR subsartorial or hunter* (Topic)
Results: 38892

Search: (regional or conduction) NEAR/2 (anaesthes* or anesthes*) (Topic) OR (nerve or conduction) NEAR/2 block* (Topic) OR "nerve infiltration" (Topic)
Results: 47641

Search: TS=(knee NEAR/2 (arthroplasty or replacement*)) OR TS=(TKA)
Results: 64019
