# Supplementary material for: Extending the Window: A Systematic Review of Pharmacological Adjuncts for Single-Shot Adductor Canal Blocks in Total Knee Arthroplasty
Source: J Clin Med. 2026 Jun 26;15(13):5005. doi: 10.3390/jcm15135005 (PMC13363162; doi:10.3390/jcm15135005)
Supplement: Supplementary file 1 [file jcm-15-05005-s001.zip › PRISMA Checklist.pdf]

PRISMA 2020 Checklist

Section and Topic	Item #	Checklist item	Location where item is reported
**TITLE**			
Title	1	Identify the report as a systematic review.	Title page/title
**ABSTRACT**			
Abstract	2	See the PRISMA 2020 for Abstracts checklist.	Abstract.
**INTRODUCTION**			
Rationale	3	Describe the rationale for the review in the context of existing knowledge.	Introduction, paragraphs 1–3
Objectives	4	Provide an explicit statement of the objective(s) or question(s) the review addresses.	End of Introduction & Methods and Materials opening paragraph.
**METHODS**			
Eligibility criteria	5	Specify the inclusion and exclusion criteria for the review and how studies were grouped for the syntheses.	Methods and Materials, “Eligibility Criteria”; grouped narratively by adjunct type and outcomes in “Data Synthesis and GRADE.”[
Information sources	6	Specify all databases, registers, websites, organisations, reference lists and other sources searched or consulted to identify studies. Specify the date when each source was last searched or consulted.	Methods and Materials, “Search Strategy” reports Medline Ovid, Embase Elsevier, CINAHL EBSCO, Cochrane CENTRAL, Web of Science Clarivate, and manual screening of reference lists
Search strategy	7	Present the full search strategies for all databases, registers and websites, including any filters and limits used.	Reported in “Search Strategy” (conference abstracts removed from Embase; registry records excluded from CENTRAL; no date, language, or study-type restrictions stated initially).
Selection process	8	Specify the methods used to decide whether a study met the inclusion criteria of the review, including how many reviewers screened each record and each report retrieved, whether they worked independently, and if applicable, details of automation tools used in the process.	“Study Selection” describes title/abstract screening followed by full-text review and manual reference screening. One reviewer worked independently, and reviewed by a second reviewer.
Data collection process	9	Specify the methods used to collect data from reports, including how many reviewers collected data from each report, whether they worked independently, any processes for obtaining or confirming data from study investigators, and if applicable, details of automation tools used in the process.	“Study Selection”
Data items	10a	List and define all outcomes for which data were sought. Specify whether all results that were compatible with each outcome domain in each study were sought (e.g. for all measures, time points, analyses), and if not, the methods used to decide which results to collect.	“Primary and Secondary Outcomes.” Primary outcome: postoperative analgesia measured using pain scores, time to first rescue analgesia, opioid consumption, or sensory block duration. Secondary outcomes: functional recovery measures and adverse events
	10b	List and define all other variables for which data were sought (e.g. participant and intervention characteristics, funding sources). Describe any assumptions made about any missing or unclear information.	Participant/intervention characteristics are reflected in Table 3 (study design, sample size, adjunct dose, local anaesthetic, comparator, primary outcomes, overall RoB)
Study risk of bias assessment	11	Specify the methods used to assess risk of bias in the included studies, including details of the tool(s) used, how many reviewers assessed each study and whether they worked independently, and if applicable, details of automation tools used in the process.	“Risk of Bias Assessment” states the Cochrane RoB 2 tool was used and results are shown in Table 1
Effect measures	12	Specify for each outcome the effect measure(s) (e.g. risk ratio, mean difference) used in the synthesis or	Outcomes are presented narratively using study-reported

Section and Topic	Item #	Checklist item	Location where item is reported
		presentation of results.	measures such as VAS/NRS pain scores, time to first rescue analgesia, opioid consumption in study-specific units, and sensory block duration; no pooled effect measure was used because no meta-analysis was performed
Synthesis methods	13a	Describe the processes used to decide which studies were eligible for each synthesis (e.g. tabulating the study intervention characteristics and comparing against the planned groups for each synthesis (item #5)).	"Data Synthesis and GRADE" indicates studies were synthesised narratively because of clinical and methodological variability and findings were categorised by adjunct type and outcomes
	13b	Describe any methods required to prepare the data for presentation or synthesis, such as handling of missing summary statistics, or data conversions.	The manuscript notes variation in study-specific units and states that direct quantitative comparison between studies was not appropriate for opioid outcomes. The review also states that pain scores are subjective and are influenced by individual patient factors.
	13c	Describe any methods used to tabulate or visually display results of individual studies and syntheses.	Results are displayed in a PRISMA flow diagram (Figure 1), RoB table (Table 1), GRADE summary table (Table 2), characteristics table (Table 3), and outcome tables (Tables 4–9).[
	13d	Describe any methods used to synthesize results and provide a rationale for the choice(s). If meta-analysis was performed, describe the model(s), method(s) to identify the presence and extent of statistical heterogeneity, and software package(s) used.	"Data Synthesis and GRADE" states that meta-analysis was not feasible because of clinical and methodological variability, so findings were synthesised narratively and presented descriptively
	13e	Describe any methods used to explore possible causes of heterogeneity among study results (e.g. subgroup analysis, meta-regression).	No formal heterogeneity analysis method is reported. Possible causes of heterogeneity are discussed narratively in the Discussion (e.g., local anaesthetic variability, ACB timing, multimodal analgesia ceiling effect)
	13f	Describe any sensitivity analyses conducted to assess robustness of the synthesized results.	No sensitivity analyses reported
Reporting bias assessment	14	Describe any methods used to assess risk of bias due to missing results in a synthesis (arising from reporting biases).	Risk of bias was discussed in the Risk of Bias Assessment and displayed in Table 1 (RoB2 tool). Publication bias is included as a GRADE domain in Table 2.
Certainty assessment	15	Describe any methods used to assess certainty (or confidence) in the body of evidence for an outcome.	"Data Synthesis and GRADE" describes use of the GRADE framework across five domains: risk of bias, inconsistency, indirectness, imprecision, and publication bias.
**RESULTS**			
Study selection	16a	Describe the results of the search and selection process, from the number of records identified in the search to the number of studies included in the review, ideally using a flow diagram.	"Study Selection" states the process is summarised in the PRISMA flow diagram (Figure 1); Results states that nine RCTs were included
	16b	Cite studies that might appear to meet the inclusion criteria, but which were excluded, and explain why they were excluded.	Discussed in Eligibility Criteria.

PRISMA

PRISMA 2020 Checklist

Section and Topic	Item #	Checklist item	Location where item is reported
Study characteristics	17	Cite each included study and present its characteristics.	Table 3
Risk of bias in studies	18	Present assessments of risk of bias for each included study.	Table 1 and accompanying "Risk of Bias Assessment"
Results of individual studies	19	For all outcomes, present, for each study: (a) summary statistics for each group (where appropriate) and (b) an effect estimate and its precision (e.g. confidence/credible interval), ideally using structured tables or plots.	Tables 4–9 present group results and significance findings for pain scores, time to first rescue analgesia, opioid consumption, sensory block duration, functional outcomes, and adverse events.
Results of syntheses	20a	For each synthesis, briefly summarise the characteristics and risk of bias among contributing studies.	Results section by outcome plus Table 3 and Table 1; broader synthesis certainty summarised in Table 2
	20b	Present results of all statistical syntheses conducted. If meta-analysis was done, present for each the summary estimate and its precision (e.g. confidence/credible interval) and measures of statistical heterogeneity. If comparing groups, describe the direction of the effect.	No statistical synthesis/meta-analysis was conducted; narrative synthesis results are reported across the Results section and Tables 4–9
	20c	Present results of all investigations of possible causes of heterogeneity among study results.	Heterogeneity is discussed narratively in the Discussion
	20d	Present results of all sensitivity analyses conducted to assess the robustness of the synthesized results.	No sensitivity analyses reported
Reporting biases	21	Present assessments of risk of bias due to missing results (arising from reporting biases) for each synthesis assessed.	GRADE Table 2
Certainty of evidence	22	Present assessments of certainty (or confidence) in the body of evidence for each outcome assessed.	Table 2, GRADE Summary of Findings
**DISCUSSION**			
Discussion	23a	Provide a general interpretation of the results in the context of other evidence.	Discussion, opening paragraphs and adjunct-specific interpretation
	23b	Discuss any limitations of the evidence included in the review.	Discussion sections addressing heterogeneity, outcome measurement gaps, functional outcomes, and limited single-study evidence for some adjuncts; also "Limitations."
	23c	Discuss any limitations of the review processes used.	Discussion/Limitations addresses heterogeneity and challenges in comparing studies.
	23d	Discuss implications of the results for practice, policy, and future research.	"Clinical Implications," later Discussion paragraphs, and Conclusion
**OTHER INFORMATION**			
Registration and protocol	24a	Provide registration information for the review, including register name and registration number, or state that the review was not registered.	Registered with PROSPERO. ID number 1384962.
	24b	Indicate where the review protocol can be accessed, or state that a protocol was not prepared.	PROSPERO.
	24c	Describe and explain any amendments to information provided at registration or in the protocol.	Not amendments made to information provided at registration or in the protocol.
Support	25	Describe sources of financial or non-financial support for the review, and the role of the funders or sponsors in the review.	Funding: "This research received no external funding." Acknowledgments also note support from the librarians

PRISMA 2020 Checklist

Section and Topic	Item #	Checklist item	Location where item is reported
			of Gold Coast Hospital and Health Service
Competing interests	26	Declare any competing interests of review authors.	"Conflicts of Interest: The authors declare no conflict of interest."
Availability of data, code and other materials	27	Report which of the following are publicly available and where they can be found: template data collection forms; data extracted from included studies; data used for all analyses; analytic code; any other materials used in the review.	"Data Availability Statement: Data is contained within the article." No separate statement about forms, extracted dataset, or analytic code availability is reported

From: Page MJ, McKenzie JE, Bossuyt PM, Boutron I, Hoffmann TC, Mulrow CD, et al. The PRISMA 2020 statement: an updated guideline for reporting systematic reviews. BMJ 2021;372:n71. doi: 10.1136/bmj.n71.
This work is licensed under CC BY 4.0. To view a copy of this license, visit https://creativecommons.org/licenses/by/4.0/
